# Multitask Learning for Activity Detection in Neovascular Age-Related Macular Degeneration

**DOI:** 10.1167/tvst.12.4.12

**Published:** 2023-04-13

**Authors:** Murat Seçkin Ayhan, Hanna Faber, Laura Kühlewein, Werner Inhoffen, Gulnar Aliyeva, Focke Ziemssen, Philipp Berens

**Affiliations:** 1 Institute for Ophthalmic Research, University of Tübingen, Tübingen, Germany; 2 University Eye Clinic, University of Tübingen, Tübingen, Germany; 3 University Eye Clinic, University of Leipzig, Leipzig, Germany; 4 Tübingen AI Center, Tübingen, Germany; 5 Hertie Institute for AI in Brain Health, University of Tübingen, Tübingen, Germany

**Keywords:** machine-learning, age-related macular degeneration, anti-vascular endothelial growth factor (VEGF) treatment, multitask learning, saliency maps

## Abstract

**Purpose:**

The purpose of this study was to provide a comparison of performance and explainability of a multitask convolutional deep neuronal network to single-task networks for activity detection in neovascular age-related macular degeneration (nAMD).

**Methods:**

From 70 patients (46 women and 24 men) who attended the University Eye Hospital Tübingen, 3762 optical coherence tomography B-scans (right eye = 2011 and left eye = 1751) were acquired with Heidelberg Spectralis, Heidelberg, Germany. B-scans were graded by a retina specialist and an ophthalmology resident, and then used to develop a multitask deep learning model to predict disease activity in neovascular age-related macular degeneration along with the presence of sub- and intraretinal fluid. We used performance metrics for comparison to single-task networks and visualized the deep neural network (DNN)-based decision with t-distributed stochastic neighbor embedding and clinically validated saliency mapping techniques.

**Results:**

The multitask model surpassed single-task networks in accuracy for activity detection (94.2% vs. 91.2%). The area under the curve of the receiver operating curve was 0.984 for the multitask model versus 0.974 for the single-task model. Furthermore, compared to single-task networks, visualizations via t-distributed stochastic neighbor embedding and saliency maps highlighted that multitask networks’ decisions for activity detection in neovascular age-related macular degeneration were highly consistent with the presence of both sub- and intraretinal fluid.

**Conclusions:**

Multitask learning increases the performance of neuronal networks for predicting disease activity, while providing clinicians with an easily accessible decision control, which resembles human reasoning.

**Translational Relevance:**

By improving nAMD activity detection performance and transparency of automated decisions, multitask DNNs can support the translation of machine learning research into clinical decision support systems for nAMD activity detection.

## Introduction

**Figure 1.**
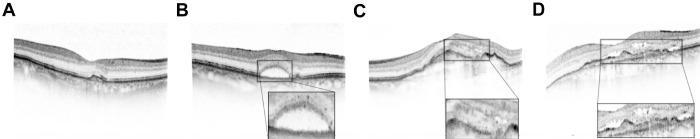
Exemplary retinal images (B-scans) with neovascular age-related macular degeneration (nAMD). (**A**) No nAMD activity. (**B**) nAMD activity due to subretinal fluid (SRF). (**C**) nAMD activity due to intraretinal fluid (IRF). (**D**) nAMD activity due to both SRF and IRF.

Neovascular age-related macular degeneration (nAMD) is a sight-threatening disease and a common cause of vision loss worldwide.^1^^–^^3^ Among the basic features of nAMD are subretinal fluid (SRF) and intraretinal fluid (IRF), which serve as surrogate markers of nAMD activity and can be monitored using optical coherence tomography (OCT^4^^,^^5^; Fig. 1).

In nAMD, increased levels of vascular endothelial growth factor (VEGF) lead to formation of new vessels from the choroidal and/or retinal vasculature. If leakage from these vessels exceeds local clearance rates, fluid builds up, leading to IRF and SRF.^4^ IRF is assumed to originate from vascular leakage from intraretinal neovascularization and/or retinal vasculature or from diffusion through the outer retina due to changes within the external limiting membrane.^4^ In contrast, SRF formation likely results from malfunction of the retinal pigment epithelium with reduced removal rates.^4^ Due to the partially different pathophysiology, IRF and SRF can occur both simultaneously and independently from each other.^4^^,^^6^ In addition, the characterization of the lesion based on IRF and SRF could help to determine the visual outcome.^7^

Treatment with intravitreal anti-VEGF agents efficiently restores the balance between fluid formation and retinal removal and is standard of care, when IRF or SRF in nAMD is detected via OCT.^5^ Prompt treatment initiation is necessary to prevent vision loss.^8^^–^^10^ Additionally, this chronic disease demands highly frequent therapy monitoring, which has put considerable burden on patients, their families, and ophthalmological care since its initial approval in 2006.^11^^–^^14^ Because the number of patients suffering from age-related macular degeneration (AMD) is thought to rise from 196 million in 2020 to 288 million in 2040, the care needed will also rise.^2^ Hence, automated solutions making the diagnostic processes more efficient have considerable appeal. For example, deep neural networks (DNNs) have been used for automatic referral decisions^15^ and predicting disease conversion to nAMD.^16^ Automated algorithms could detect both SRF and IRF more reliably than retinal specialists, especially in less conspicuous cases.^17^ Ideally, such automated tools serve to support retinal specialists in their decision making. In collaboration, a retina specialist assisted by an artificial intelligence (AI) tool can outperform the model alone (e.g. for the task of diabetic retinopathy grading).^18^ To this end, computational tools need to explain their decisions and communicate their uncertainty to the treating ophthalmologist.^19^^,^^20^

**Figure 2.**
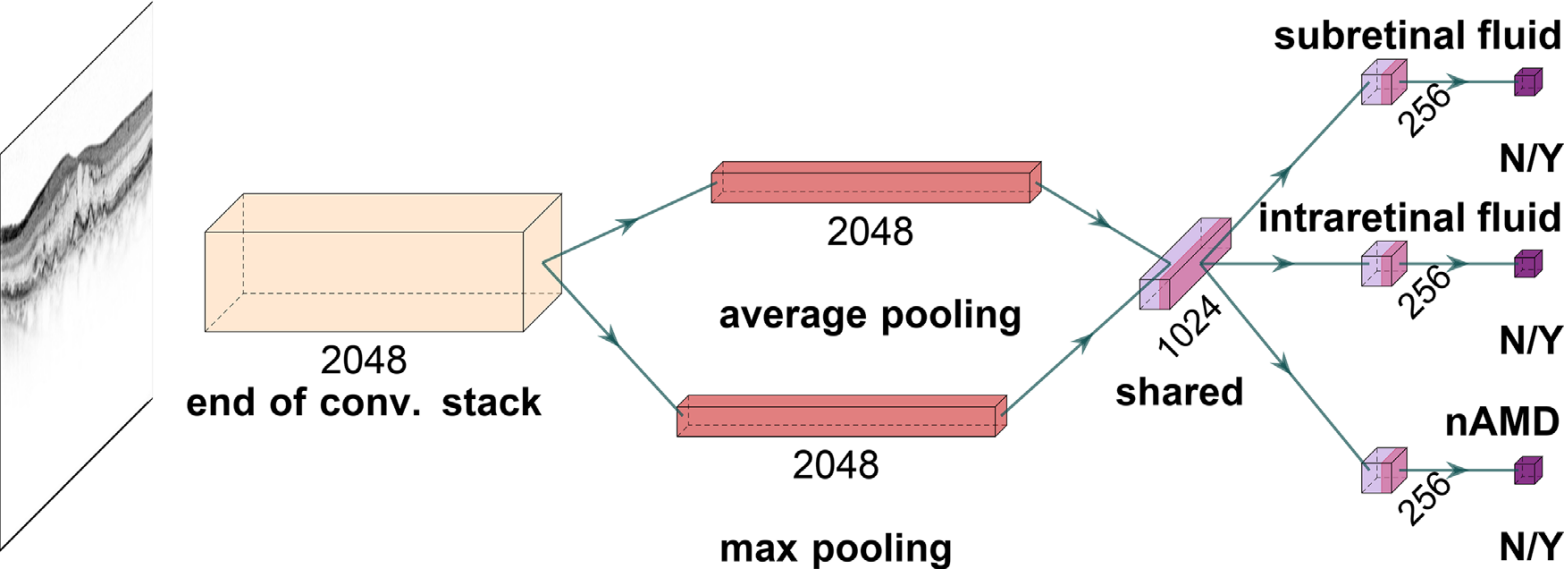
A deep neural network for simultaneous detection of subretinal and intraretinal fluid as well as the nAMD activity from OCT B-scans. Given a B-scan, convolutional stack of the InceptionV3 architecture extracts 2048 feature maps. These are average and max pooled, and fed into a fully connected (dense) layer with 1024 units for shared representation. Then, task-specific heads specialize into individual tasks and single units with sigmoid function achieve binary classification based on 256 task-specific features.

Here, we develop a convolutional deep learning model based on the concept of multitask learning.^21^^,^^22^ Multitask learning is a generalization of the widely used single-task learning, where models are trained for multiple input-output mappings simultaneously (Fig. 2). For instance, multitask models can be used to capture different characteristics of dry AMD, such as drusen area, geographic atrophy, increased pigment, and depigmentation, to combine these outputs into final AMD diagnosis with respect to a nine-step severity scale.^23^ Multitask learning has also shown prognostic value when applied to survival analysis via two simultaneous prediction tasks: drusen and pigmentation grading.^24^ In a similar vein, our multitask model detects SRF, IRF, and nAMD activities in parallel. However, it generates distinct outputs for each of these tasks and offers well-calibrated uncertainty estimates for each of them, which is unique to our study. As the fluid compartment plays a decisive role in the treatment outcome^25^^–^^27^ with the simultaneous presence of IRF and SRF being associated with the worst prognosis,^9^ we visualize the representation driving the DNN-based decisions using t-distributed stochastic neighbor embedding (t-SNE)^28^^,^^29^ and investigate the model's decisions using clinically validated saliency mapping techniques.^30^ Thus, together with well-calibrated uncertainty reports, our work provides an interpretable tool for the ophthalmologist to rapidly access the neural network's decision process on both population-based and individual-patient levels as a prerequisite for clinical application.

## Methods

### Data Collection

This study included 70 patients (46 women and 24 men) with nAMD at least in one eye, seen by an ophthalmology resident (author A.G.) in the Macula clinic at the University Eye Hospital Tübingen. Exclusion criteria were any other cause of neovascularization, any co-existing retinal pathology (e.g. epiretinal membrane, macular hole, and diabetic retinopathy), glaucoma, and media opacity preventing sufficient image quality. There were 3762 B-scans (2011 of the right eyes and 1751 of the left eyes) of 440 × 512 pixels taken with the Heidelberg Spectralis OCT (Heidelberg Engineering, Heidelberg, Germany) that were included in the study. A retina specialist of the same hospital (author I.W.) assessed disease activity and presence of IRF and SRF on each individual B-scan (see Fig. 1). Disease activity was also graded by a resident (author A.G.). B-scans were assigned to a training, validation, or test set (Table 1). All images of one patient were assigned to one set to avoid information leakage. The study was conducted in accordance with the tenets of the Declaration of Helsinki and approved by the local institutional ethics committee of the University of Tübingen, which waived the requirement for patient consent due to the study's retrospective characteristics.

**Table 1.** OCT Data Distribution of Subretinal Fluid (SRF), Intraretinal Fluid (IRF) and Active nAMD in B-Scans in Training, Validation, and Test Sets, Respectively. Absolute and Relative Numbers Are Shown

	Training			Validation			Test		
	Subretinal Fluid	Intraretinal Fluid	Active nAMD	Subretinal Fluid	Intraretinal Fluid	Active nAMD	Subretinal Fluid	Intraretinal Fluid	Active nAMD
***Yes***	639	286	848	69	58	101	161	153	269
	(0.232)	(0.104)	(0.308)	(0.170)	(0.143)	(0.248)	(0.267)	(0.253)	(0.445)
***No***	2112	2465	1903	338	349	306	443	451	335
	(0.768)	(0.896)	(0.692)	(0.830)	(0.857)	(0.752)	(0.733)	(0.747)	(0.555)

### Diagnostic Tasks, Network Architecture, and Model Development

We developed a multitask DNN to detect the presence of SRF and IRF as well as the nAMD activity from OCT B-scans (see Fig. 2). As the backbone, we used the InceptionV3 architecture^31^ via Keras,^32^ which was pretrained on ImageNet^33^ for 1000-way classification via a “softmax” function. We used the InceptionV3 DNN's convolutional stack as is but linked max pooling and average pooling layers to the end of convolutional stack and concatenated their outputs to obtain 4096-dimensional feature vectors. These were followed by a dense layer, which yielded a shared representation with 1024 features. To this, we added task-specific heads with 256 units, which specialized into their respective tasks. Then, task-specific binary decisions were achieved by single units equipped with sigmoid functions. For training, our DNNs in both single and multitask scenarios, we resorted to the retina specialist's set of labels.

We trained our networks with equally weighted cross-entropy losses for all tasks on the training images: *D* = {**x**_n_, **y**_n_}, n = 1,…,N, where **y**_n_ was a vector of binary labels indicating nAMD activity and the presence of IRF or SRF in an image **x**_n_. Parameterized by θ, a DNN f_θ_(·) was optimized with respect to the total cross-entropy on the training data:

(1) $$\begin{array}{c} L\left(D,f_{\theta }\left(\cdot \right)\right)=\frac{1}{N}\sum_{n=1}^{N}l\left(y_{n},\;f_{\theta }\left(x_{n}\right)\right), \end{array}$$

where

(2) $$\begin{array}{ccc} & & l(y_{n},\;f_{\theta }(x_{n}))=\sum_{t=1}^{T}y_{n,t}\;\log\;p_{n,t}\\ & & +\;(1-y_{n,t})\;\log(1-p_{n,t}), \end{array}$$

*p_n,t_* was a probability estimated via the sigmoid function for a task indicated by *t*, and *T* was the total number of tasks. For *T* = 1, multitask learning was reduced to single-task learning based on the same architecture but with only one task head. We also developed a two-task model to perform the SRF and IRF detection tasks (*T* = 2), whereas eliminating the redundancy of the nAMD activity detection task, which is, in principle, a function of the former two. To address the class imbalance (see Table 1), we used random oversampling (see the Quantification of uncertainty via *mixup* and Deep Ensembles heading for details). We trained the DNN using Stochastic Gradient Descent (SGD) with Nesterov's Accelerated Gradients (NAG),^34^^,^^35^ minibatch size of 8, a momentum coefficient of 0*.*9, an initial learning rate of 5 · 10^−4^, a decay rate of 10^−6^, and a regularization constant of 10^−5^ for 120 or 150 epochs (see the Data augmentation and preprocessing heading for longer training). During the first five epochs, the convolutional stack was frozen and only dense layers were trained. Then, all layers were fine-tuned to all tasks. The best models were selected based on total validation loss after each epoch and used for inference on the test set.

#### Data Augmentation and Preprocessing

We used Mixup^36^ for data augmentation during training. Mixup generates artificial examples through the convex combinations of randomly sampled data points. We adapted Mixup to our multitask learning scenario as follows:

(3) $$\begin{array}{ccc} \hat{x} & = & \lambda x_{i}+\left(1-\lambda \right)\;x_{j},\\ \hat{y} & = & \lambda y_{i}+\left(1-\lambda \right)y_{j},\;\;\lambda \in \left[0,1\right]. \end{array}$$

Mixing was controlled by *λ* ∼ *Beta*(*α, α*), where *α* ∈ (0*, ∞*). For *α* = 0, *λ* is either 0 or 1, and there is no mixing. We used 0, 0.05, 0.1, and 0.2 for *α* and trained networks for 120 epochs when not mixing and 150 epochs when mixing. In addition, to allow for a warm-up period when mixing,^36^ we set *α* = 0 for the first five epochs. In addition, we applied common data augmentation operations, such as adjustment of brightness within ±10%, horizontal and vertical flipping, up and down scaling within ±10%, translation of pixels horizontally and vertically within ±30 positions, and random rotation within ±45 degrees. After all data augmentation operations, we used an appropriate preprocessing function (keras.applications.inception_v3.preprocess_input.) from the Keras API.^32^

#### Quantification of Uncertainty Via Mixup and Deep Ensembles

DNNs often do not generate well-calibrated and reliable uncertainty estimates for their decision.^37^^–^^41^ However, quantification of diagnostic uncertainty is crucial for treatment decisions because proper management can minimize diagnostic errors, delays, or excess healthcare utilization.^42^ Mixup^36^ improves the calibration of DNN outputs by smoothing labels through their convex combinations (Equation 3).^43^ In addition, we used Deep Ensembles^39^ consisting of multiple DNNs with different random initializations.^39^^,^^44^ This can improve upon the single network performance both in accuracy and calibration, even with small numbers of DNNs.^39^^,^^44^^–^^46^ We used ensembles with three DNNs, for which we enforced diversity by a specialized oversampling strategy: for each DNN, we oversampled training images with respect to one of the task's labels. This enabled DNNs to train on a balanced dataset while also learning about other tasks, even though the data were not balanced for these. We then used the ensemble's mean output for predictions and quantified uncertainty in terms of entropy, given the average predictive probabilities.

### Low-Dimensional Embedding of Images

We used t-SNE^28^ to obtain further insights into the decision-making process of our ensemble model. The t-SNE is a nonlinear dimensionality reduction method, that embeds high-dimensional data points into a low-dimensional space. We concatenated features from ensemble members’ predetermined read-out layers and performed t-SNE based on them, embedding each B-scan into the two-dimensional plane. We used openTSNE^47^ with PCA initialization to better preserve the global structure of the data and improve the reproducibility.^29^ A perplexity of 200 for 1500 iterations with an early exaggeration coefficient of 12 for the first 500 iterations was used according to best-practice strategies.^29^ Similarities between data points were measured by Euclidean distance in the feature space.

### Saliency Maps

We used Layer-wise Relevance Propagation (LRP)^48^ to compute saliency maps highlighting the regions in the OCT images which contributed to the DNN decisions, as it provides most clinically relevant tasks.^30^ We created three saliency maps for each OCT slice: subretinal, intraretinal, and disease activity in nAMD. To improve the visualization of the salient regions, saliency maps were postprocessed.^30^ Saliency maps were only shown for predictions with an estimated probability greater than 0*.*5, because, as previous work has shown, that especially in absence of disease, saliency maps can lead physicians to overdiagnosis.^18^

## Results

**Figure 3.**
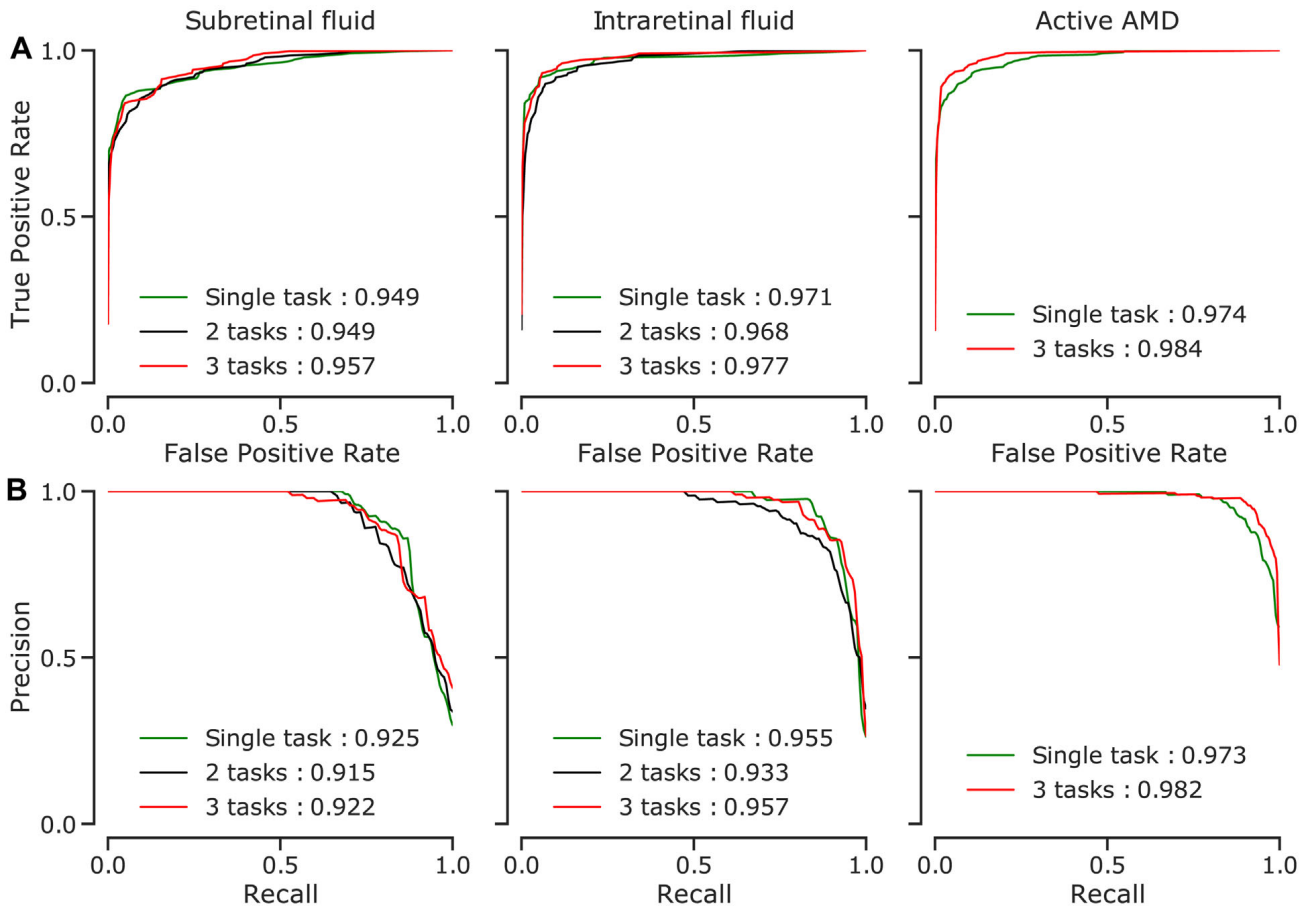
Performance curves of the selected models on the test images. Area under the curve (AUC) values given for models also summarize the overall performance into one number (higher is better). (**A**) Receiver Operating Characteristics (ROC) curves. (**B**) Precision-recall curves.

**Figure 4.**
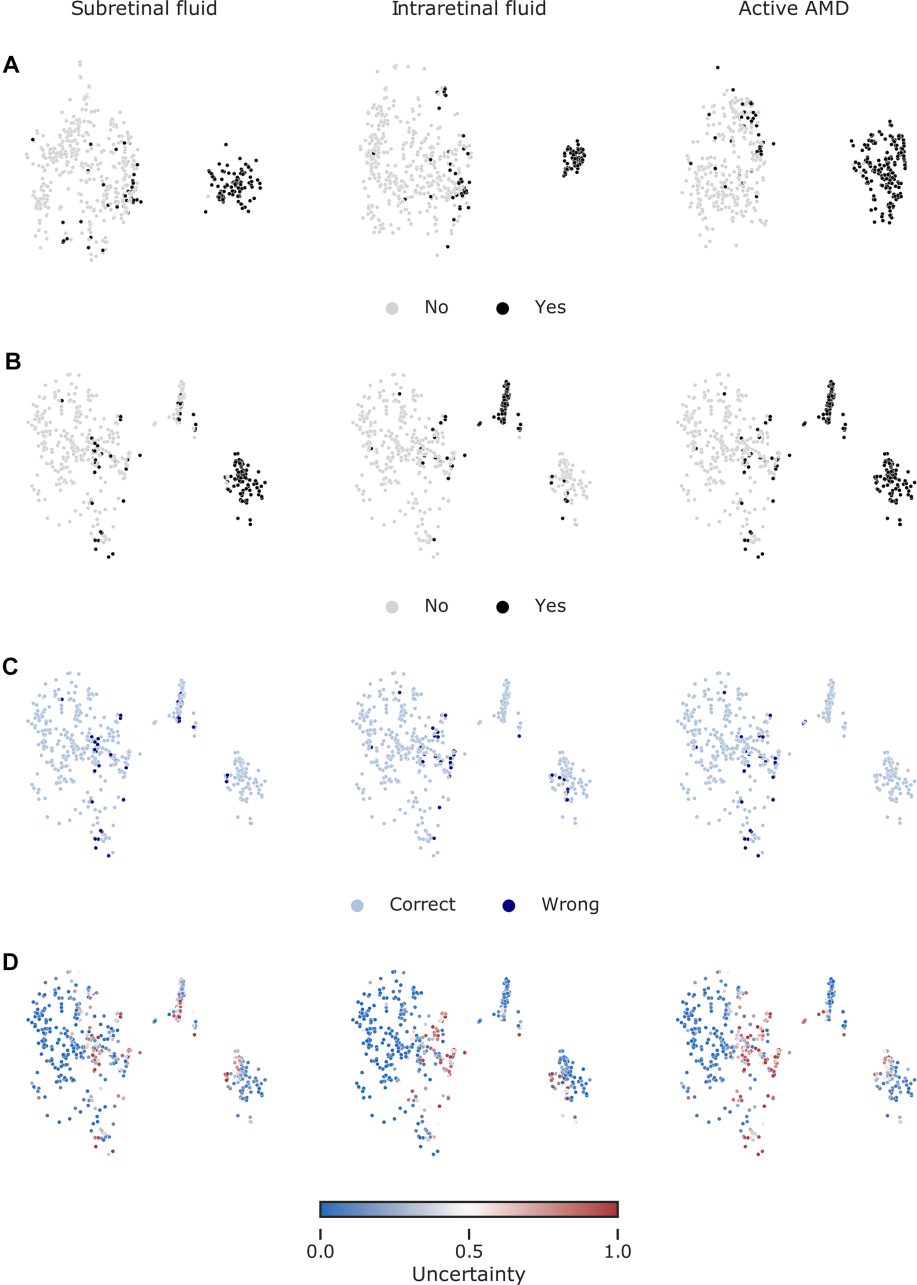
Visualization of data via t-SNE of ensemble-based representations. Only the test data are shown. (**A**) Low dimensional embedding of images based on the 1024-dimensional features from the pre-penultimate layers of single-task networks. Colored with respect to the task-specific labels. (**B**) Same as in **A** but with respect to 1024 features from the shared representation layer of multitask networks. (**C**) Same map as in **B** but colored with respect to correct and wrong predictions. (**D**) Same map as in **B** but colored with respect to uncertainty minimum-maximum normalized to [0, 1].

We developed an ensemble of three multitask DNNs to simultaneously detect SRF, IRF, and activity of nAMD on OCT B-scans (see Fig. 1). Each DNN consisted of a shared convolutional core combined with pooling operations and a fully connected (dense) layer (see Fig. 2). The resulting shared representation served as the basis for the decisions of the three task-specific heads. The idea behind this approach is that the DNN can benefit from the shared representation induced by combining information from different tasks. We compared the performance of the multitask model with more specialized single-task models, where we constructed three DNNs for each task, which did not share any representation but were trained independently. In addition, we also used a two-task model that simultaneously detected only SRF and IRF, without the nAMD activity detection head. All DNNs were trained on the same dataset (see Table 1 and Methods), which was graded according to the nAMD activity by a retina specialist (author I.W.) and an ophthalmologist resident (author A.G.) with high intergrader agreement on disease activity (Cohen's kappa = 0.86). In a second step, the retina specialist further examined the data for the presence of IRF and SRF. The two retinal fluid types occurred largely independently, whereas there was natural overlap of both with the active AMD label (Table 2). We selected the three-task model with the best accuracy for the activity detection task on the validation set and report accuracy values computed on an independent test set (Table 3). The three-task model was well calibrated on the test set (Adaptive expected calibration error^41^ of 0.0147 for SRF, 0.0104 for IRF, and 0.0263 for active nAMD). We found that the performance of the 3-task model surpassed the single-task model performance in disease activity detection, reaching an accuracy of 94.2% for the multitask model versus 91.4% for the single task model (Table 3, Fig. 3). This three-task model optimized for AMD activity detection performed slightly worse than the single-task models for SRF and IRF detection (SRF: accuracy of 0.917 vs. 0.924 for multitask versus single-task; IRF = 0.937 vs. 0.950). For the two-task scenario, we selected the model with the highest average validation accuracy across the SRF and IRF detection tasks. Interestingly, the two-task model performed worse than the single-task and three-task models. This highlights the importance of the explicit nAMD activity detection head in the three-task model. We then further studied the representations learned by the models to gain insight into their decision making process. To this end, we extracted the representations of individual OCT scans from both single-task and multitask models and created two-dimensional embeddings of these via t-SNE (Fig. 4). In these visualizations, each point represents an individual OCT scan. Scans which are similar to each other according to the learned representation are mapped to nearby points. Of note, distances and, in particular, the size of white space between clusters in t-SNE plots should be carefully interpreted.^29^^,^^49^

**Table 2.** Agreement of Task-Specific Labels Across Training, Validation and Test Sets, Measured via Cohen's Kappa Statistic, Which Is Essentially a Number Between −1 and 1. Whereas 1 Indicates a Full Agreement, Lower Scores Mean Less Agreement. Negative Scores Indicate Disagreement

	Training			Validation			Test		
	Subretinal Fluid	Intraretinal Fluid	Active nAMD	Subretinal Fluid	Intraretinal Fluid	Active nAMD	Subretinal Fluid	Intraretinal Fluid	Active nAMD
**Subretinal fluid**	–	−0.02	0.79	–	0.26	0.75	–	−0.02	0.59
**Intraretinal** **fluid**	−0.02	–	0.37	0.26	–	0.65	−0.02	–	0.57

**Table 3.** Accuracy of Ensembles for Various Degrees of Mixing (Indicated by α). Gray Row Indicates the Ensemble of Choice for Further Analysis Based on the Validation Performance for the Activity Detection Task. In the Two-Task Scenario, The Average Validation Accuracy of SRF and IRF Detection Tasks was used for Model Selection

Training				Validation			Test		
	Subretinal Fluid	Intraretinal Fluid	Active nAMD	Subretinal Fluid	Intraretinal Fluid	Active nAMD	Subretinal Fluid	Intraretinal Fluid	Active nAMD
Single task models									
*α* = 0	1.000	1.000	1.000	0.988	0.971	0.958	0.924	0.950	0.914
*α* = 0*.*05	0.983	0.994	0.975	0.971	0.963	0.951	0.906	0.919	0.909
*α* = 0*.*1	0.978	0.994	0.948	0.948	0.919	0.929	0.868	0.891	0.856
*α* = 0*.*2	0.983	0.991	0.851	0.975	0.946	0.853	0.881	0.909	0.702
Two task models: SRF and IRF									
*α* = 0	0.999	1.000	–	0.968	0.961	–	0.902	0.937	–
*α* = 0*.*05	1.000	0.999	–	0.983	0.966	–	0.927	0.919	–
*α* = 0*.*1	0.999	0.999	–	0.983	0.973	–	0.911	0.924	–
*α* = 0*.*2	0.999	1.000	–	0.983	0.963	–	0.917	0.932	–
Three task models: SRF, IRF, and nAMD activity									
*α* = 0	1.000	0.995	0.998	0.973	0.973	0.961	0.914	0.935	0.940
*α* = 0*.*05	0.999	0.998	1.000	0.971	0.971	0.966	0.917	0.937	0.942
*α* = 0*.*1	1.000	0.997	0.998	0.983	0.968	0.966	0.916	0.957	0.939
*α* = 0*.*2	1.000	0.998	1.000	0.971	0.966	0.966	0.894	0.937	0.906

We labeled individual points according the evidence for SRF or IRF and overall AMD activity. In the single-task DNNs, well-separated clusters were found, indicating only the learned task-label (see Fig. 4A). For example, OCT scans with SRF present formed a single cluster, clearly distinct from the OCT scans without this label. In contrast, in the multitask network, subclusters within the active nAMD data points were observed (see Figs. 4A, 4B): OCT scans labeled with SRF formed a well-separated cluster at the bottom right, as did scans with IRF labels at the top right (see Fig. 4B). Interestingly, there was a small cluster in between these two which contained scans labeled with both. This suggests that multitask DNNs learned a representation which could differentiate between the two fluid types. The few incorrectly classified OCT scans could be found within their clusters to be placed close toward other clusters (see Fig. 4C) in areas where we also found examples with high classifier uncertainty (see Fig. 4D).

**Figure 5.**
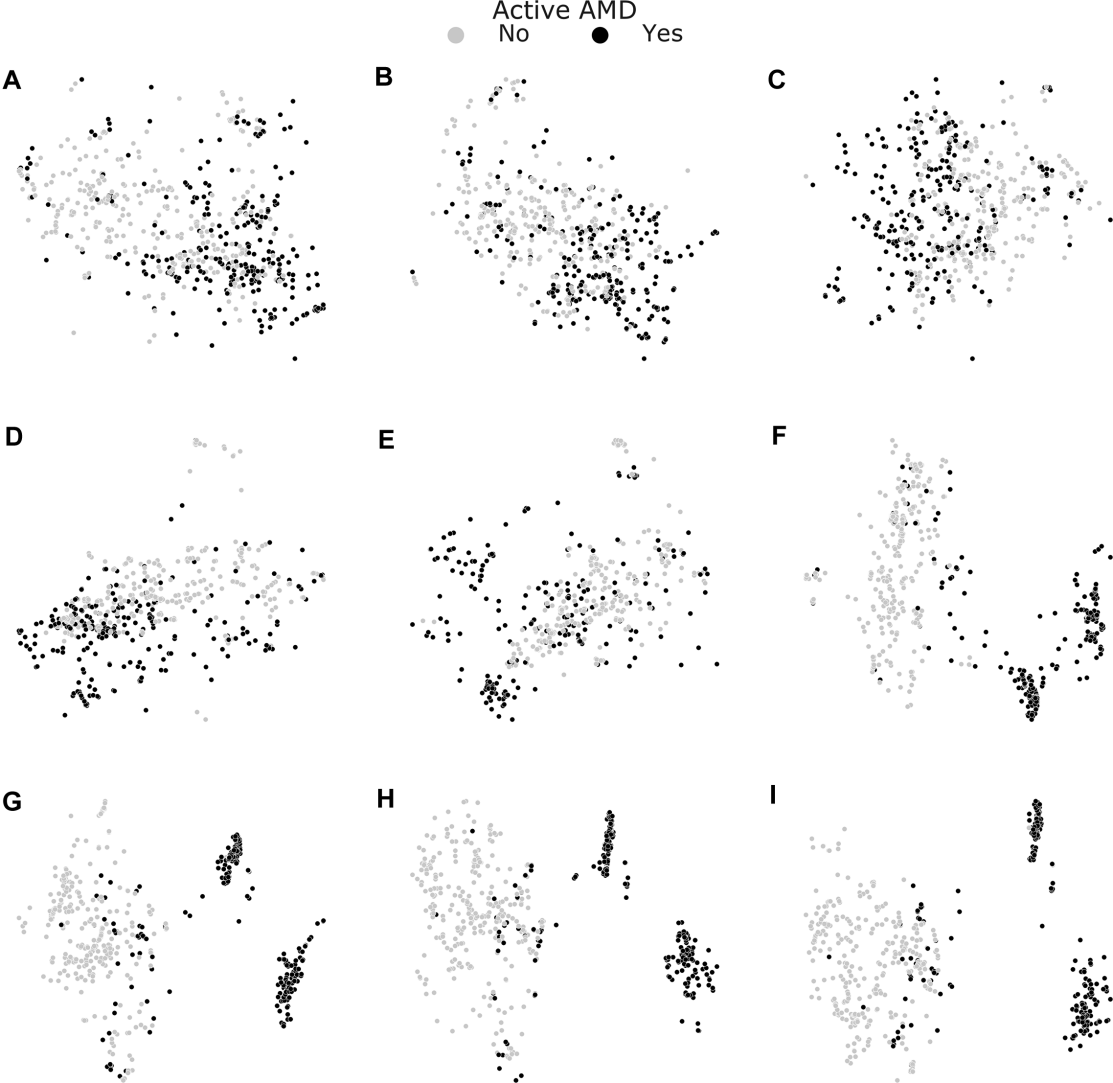
Layer-wise visualization of test data via t-SNE. Starting just before the first inception module (**A**) and reading out feature representations yielded by every other module (**B-F**) along with the last inception module (**G**), the shared representation layer (**H**) and the nAMD activity detection head's penultimate layer (**I**), we performed t-SNE with the aforementioned settings. Useful representations emerged toward the end of convolutional stack and the task-specific representation allowed the best separation of nAMD active cases from those inactive.

**Figure 6.**
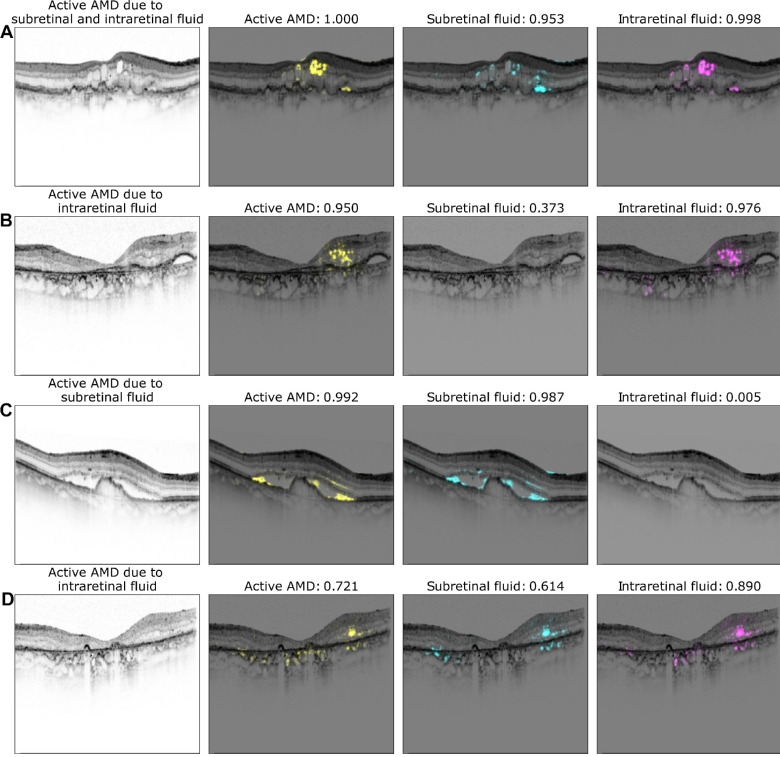
Exemplary saliency maps for four optical coherence tomography (OCT) images. The first column displays the OCT B-scan with the corresponding labeling of a retinal specialist. Second to fourth columns show saliency maps and the network's confidence for active nAMD (*yellow*), subretinal fluid (SRF; *cyan*), and intraretinal fluid (IRF; *magenta*). Note, that saliency maps are only shown in case of confidence >0.5.

**Figure 7.**
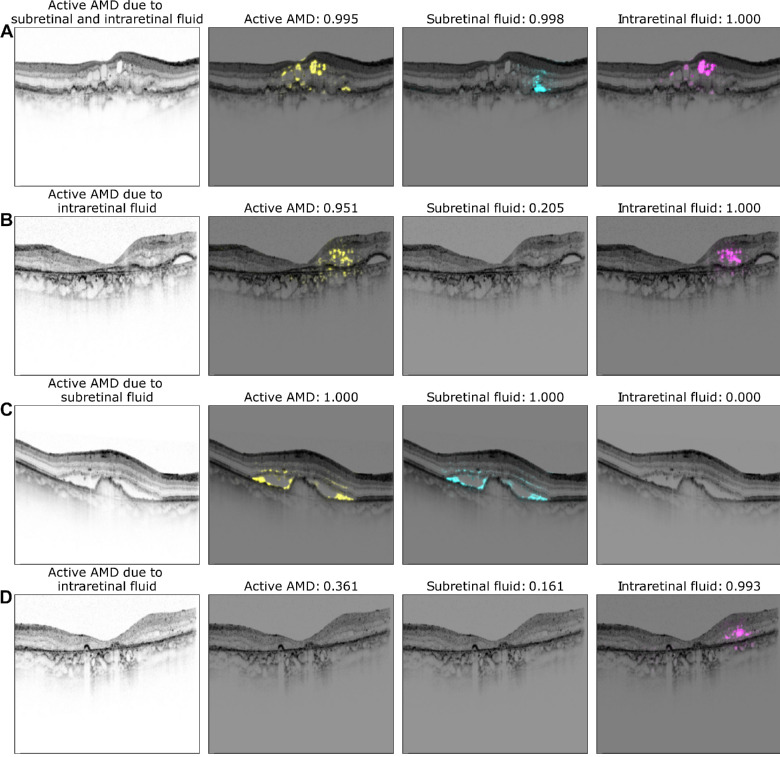
Exemplary saliency maps as in Figure 6 but results were obtained from single-task models.

We next studied how the multitask representations emerged through processing in the network (Fig. 5). Whereas in the initial layers, data points representing active nAMD were still uniformly distributed (see Figs. 5A-C), a clear separation of active nAMD cases developed gradually in later layers of the DNN (see Figs. 5D-G), leading to best separation in the shared representation (see Fig. 5H). The decision head for active AMD refined this representation only very little (see Fig. 5I). We finally analyzed the saliency maps of the multitask DNNs and asked whether the saliency maps for the subtasks of SRF and IRF detection obtained from the multitask model allowed reasoning about evidence specific to these tasks. We generated saliency maps on four exemplary OCT scans using LRP^48^ (see Fig. 6). For an OCT scan with clearly active AMD and both SRF and IRF present (see Fig. 6A), we found that the active AMD saliency map focused on intraretinal fluids, which were also clearly visible in the task-specific saliency map, and faintly highlighted regions with SRF. The SRF saliency map, however, clearly highlighted SRF. In two further example scans with either IRF or SRF, respectively, active AMD saliency maps clearly corresponded to the individual task maps (see Figs. 6B, 6C). We also identified a rare failure case of the obtained saliency maps (see Fig. 6D), where an OCT scan was falsely classified positive for SRF with a confidence of 0.614 due to the misclassification of IRF to SRF. We hypothesize that the DNN misclassified the superior border of the IRF as photoreceptor layer detached from the retinal pigment epithelium. The assumption that the DNN primarily recognizes contrast-rich interfaces, such as SRF and IRF, is further supported by the false labeling of cystoid spaces within choroid in Figures 6B and 6D, whereas in a smoother, lower-contrast choroid saliency, maps do not highlight any structures (see Fig. 6). Comparison with saliency maps from the single-task DNNs (Fig. 7) to those generated from the multitask models shows that those single-task saliency maps appear slightly more defined, but generally highlight similar areas.

## Discussion

In this study, we developed a multitask learning model to simultaneously detect SRF and IRF, as well as disease activity in OCT B-scans of patients with nAMD. We showed that a three-task model, which takes the presence of IRF and SRF into account to detect disease activity in nAMD, surpassed a single task model regarding accuracy in the activity detection task. Furthermore, our visualization of the multitask model's decision-making process via t-SNE showed that inactive and active nAMD B-scans formed different clusters. Among active AMD B-scans, three distinct clusters were observed, which contained OCT B-scans with either SRF or IRF or both fluid types. This separation could not be seen in the single-task models. Saliency maps of exemplary B-scans further corroborate that task-relevant information can be extracted from the multitask networks. Thus, multitask DNN could serve as a basis for an explainable clinical decision support system for nAMD activity, providing support for clinicians in detecting active AMD, but would also allow clinicians to identify evidence in the relevant sub-tasks of finding SRF and IRF.

Ophthalmology has recently seen a development of various AI systems, yet their use in clinical routine remains rare with only few systems available on the market.^50^^,^^51^ One big barrier is potential harm of the patient-physician relationship going hand in hand with the lack of trust in those systems.^52^ Here, we combined multitask DNNs with different visualization methods to give an insight into the DNNs’ reasoning and increase transparency. First, we used t-SNE as the visualization method for high-dimensional data^28^^,^^29^ (see Fig. 4) to present the decision-making process of the model. This form of visualization provides an intuitively interpretable rationale for how OCT B-scans were graded by visualizing which other B-scans are similar. The resulting visualization may also increase an ophthalmologist's confidence in the model because it illustrates that model's decision-making reasoning resembles their own. We further analyzed the multitask model's decision on saliency maps of individual OCT scans. Saliency maps highlight critical regions for the model's decision and thus allow a quick visual control of its reasoning. This may be important in cases of advanced AMD, where fluid is due to degeneration rather than exudation to avoid overtreatment. However, different methods of saliency map agree to differing degrees with clinical annotations^30^^,^^53^^,^^54^ and saliency maps can lead to overdiagnosis.^18^ Therefore, we used the saliency map technique with the best clinical relevance for AMD activity^30^ and displayed saliency maps in case of a confidence of the algorithm >0.5. Compared to saliency maps of single task DNNs, multitask saliency maps seem to draw slightly less sharp contours, however, we found good overlap between regions used for active AMD detection and those for SRF and IRF.

Limitations of our study are also worth considering for further research. For instance, a recent meta-analysis has provided evidence of varying influences of SRF and IRF on the visual outcome in nAMD patients.^55^ Stable SRF might not affect visual outcome, whereas fluctuations in IRF during treatment seem to negatively influence visual acuity.^55^ For this reason, treatment decisions in nAMD solely on a yes or no basis may not meet future treatment guidelines, which might rather require a sophisticated decision depending on the present fluid type and its variation in volume for or against an anti-VEGF injection. Other signs of active nAMD, such as hard exudates, pigment epithelial detachment, subretinal hyperreflective material, or hyper-reflective foci,^4^^,^^56^ can be also added to multitask decision pipelines.

Future studies will also need to assess how well these multitask learning results transfer from this data sample acquired at a tertiary center in Germany. It would be desirable to perform similar analysis with larger and more diverse data sets, to test also the generalization to other populations, and different recording qualities, as well as OCT devices (including mobile devices). Further, performance could be potentially increased by combining the multitask network with a segmentation layer,^15^ which could reduce false positive cases. Additionally, in clinical routine, activity decision is made on a whole volume not a single B-scan, which could technically be implemented by combining the results from individual B-scans (e.g. by majority voting or uncertainty propagation).

Although the approval of anti-VEGF has decreased economic and overall treatment burden of nAMD measured in disability-adjusted life,^57^^,^^58^ a large number of patients still discontinue treatment.^59^ Patients named the need for assistance, either in the form of a travel companion or a family member, as the main reason for discontinuation.^14^ Additionally, recurrence of quiescent disease requiring prompt treatment is common, making life-long monitoring necessary.^60^ For these reasons, automated solutions allowing monitoring close to home or even at home are promising technologies.^61^^,^^62^ They provide easier access and reduce the disease burden on the individual.^63^ Automated solutions for fluid detection have further gained popularity during the coronavirus disease 2019 (COVID-19) pandemic, which showed the devastating effects of delay or interruption of nAMD treatment on visual function.^9^^,^^60^ Despite promising results in laboratory settings, real-world data revealed significantly lower performance rates of home-based OCT with, in particular, SRF being overlooked by the system.^64^ This shows the necessity of further developments on the machine learning side to guarantee reliable use, with multitask learning as suggested in this study being a viable option.
